# Injury prevalence across sports: a descriptive analysis on a representative sample of the Danish population

**DOI:** 10.1186/s40621-018-0136-0

**Published:** 2018-04-02

**Authors:** A. M. Bueno, M. Pilgaard, A. Hulme, P. Forsberg, D. Ramskov, C. Damsted, R. O. Nielsen

**Affiliations:** 1 Department of Clinical Medicine, Aarhus University, Palle Juul-Jensens Boulevard 82, 8200 Aarhus, Denmark; 2 The Danish Institute of Sports Studies, Frederiksgade 78B, 8000 Aarhus, Denmark; 3 Centre of Human Factors and Sociotechnical Systems. University of the Sunshine Coast, Queensland, Australia; 4 The Danish Institute of Sports Studies, Kanonbådsvej 4A, 1437 København K, Denmark; 5 Department of Physiotherapy, University College Northern Denmark, 9000 Aalborg, Denmark; 6 Section for Sport Science, Department of Public Health, Aarhus University, Dalgas Avenue 4, 8000 Aarhus, Denmark

## Abstract

**Background:**

Physical activity plays an important role in public health, owing to a range of health-related benefits that it provides. Sports-related injuries are known to be an important barrier to continued physical activity. Still, the prevalence of injuries on a general population level has not yet been explored in a descriptive epidemiological investigation. The purpose of the questionnaire-based study, therefore, was to describe the prevalence of injury in a representative sample of the Danish population.

**Methods:**

Two samples of 10,000 adults (> 15 years) and 6500 children and adolescents (7–15 years) were invited to respond to a web-based questionnaire. Of these, 3498 adults (35.0%) and 3221 children (49.6%) responded successfully. The definition of sports injury was time-loss and medical attention-based, inhibiting participants from sports activity for at least 7 days, and/or involved contact with a healthcare professional, respectively.

**Results:**

Amongst adults, 642 (18.4% [95%CI: 17.1%; 19.6%]) reported to have had an injury within the past 12 months. Males reported significantly more injuries than females (difference in prevalence proportion: 9.2%-points [95%CI: 6.7%-points; 11.8%-points]). The prevalence of injuries was greatest in running (n_inj_ = 198), football (n_inj_ = 94) and strength training (n_inj_ = 89).

Amongst children, 621 (19.3% [95%CI: 17.9%; 20.6%]) had been injured. No difference in injury prevalence proportion existed between boys and girls. The prevalence of injuries was greatest in football (n_inj_ = 235), handball (n_inj_ = 86) and gymnastics (n_inj_ = 66).

**Conclusions:**

Sports injuries seem to be very frequent in Denmark, since a total of 18.4% of the adults and 19.3% of the children reported having had one or more injuries within the past 12 months, equal to either time lost with physical activity and/or contact to the health care system.

## Background

The health benefits associated with physical activity are well accepted in the scientific literature, particularly since physical activity plays an important role in both the prophylaxis and treatment of a number of lifestyle diseases (Klarlund & Andersen, 2011). To counteract the deleterious effect of inactivity, which is reportedly the second biggest risk factor for death in Denmark (Eriksen et al., 2016), at least 30 min of physical activity per day has been recommended for adults by The Danish Health Authority (Klarlund & Andersen, 2011). Likewise, amongst children and adolescents, a minimum level of 90 min per day of physical activity has been recommended. In 2011, the prevalence proportion of physically active children and adults in Denmark was 86% and 64%, respectively (Laub, 2013). Children aged 7–9 years had the greatest level of physical activity, whereas adults aged above 70 years had the least.

Owing to the health-related benefits from being physically active, it is important to shed light on the barriers for becoming physically active, including those that also prevent individuals from maintaining a physically active lifestyle. Various barriers exist, including a lack of motivation having limited health literacy, time constraints, or being physically impaired (Klarlund and Andersen 2011; Rosenbaum et al. 2016). Another barrier is sports injury, which can lead to a temporary or permanent break from the chosen activity of interest. Nielsen et al. (Nielsen et al., 2014) found a median time-to-recovery of almost 3 months amongst injured novice runners, thus leading to reduced health-related benefits due to less activity.

According to the TRIPP model (Finch, 2006), the first step in injury research is to understand the extend of the problem. The prevalence and prevalence proportion of sport injuries has been widely investigated across sports. Unfortunately, such studies have only included groups selected by either one or more criteria, such as specific sport (Jacobsson et al., 2012), level (Hall et al., 2013), age (Scase et al., 2012) or injury type (Maselli et al., 2015). The recruitment of selected groups has further limited the external validity of study results to the general population. In addition, knowledge about the prevalence of sports injuries on a general population level is, alongside injury severity and treatment costs, important in order to identify whether sports injuries are a public health burden, as well as to identify whether certain sports contribute to a larger number of injuries than others (Finch, 2006). To our knowledge, no studies have yet investigated the total prevalence of sport injuries in a general population-based sample, and subsequently compared the prevalence and prevalence proportion of sports injuries between different sports.

Therefore, the primary aim of this study was to add to the literature the prevalence proportion of sport injuries in a representative sample of the general Danish population. The secondary aim was to describe the prevalence and prevalence proportion of injuries in different sports.

## Methods

### Design

The study was designed as a questionnaire-based study. The Danish Data Protection Agency approved the study and in accordance with Danish law, approval from the local ethics committee is only required in studies with an intervention. In primo January 2016, questionnaires were distributed via postal mail by a local company (Rambøll, Denmark, using SurveyXact) to a representative sample of the general Danish population.

### Sampling

The Danish Civil Registration System (CRS), an administrative register established on April 2, 1968, which contains individual-level information on all persons residing in Denmark (and Greenland as of May 1, 1972), was used to identify: (i) a sample of adults consisting of 10.000 persons above 15 years; and, (ii) a sample of children and adolescents consisting of 6.500 persons between 7 and 15 years. A unique ten-digit Civil Personal Register number was assigned to all persons in the CRS which allowed for the identifications of birth date and gender (Schmidt et al., 2014). All 10.000 adults and 6.500 children were randomly selected from CRS.

### Data-collection

The study sample was provided with a written letter by postal mail. For the children and adolescents, the letter was forwarded to the parent who was encouraged to help with the questionnaire. The letter contained a short introductory text about the survey history, method and aim, and contained a person-specific code, which was then used to access a web-based questionnaire. The recipients were encouraged to access the web-based questionnaire through a standard tablet or computer, and informed to complete all questions.

In January 2016, non-responders received a reminder by postal mail. In cases of no response, they were contacted by phone. In March, they received a second postal mail reminder.

**Fig. 1:**
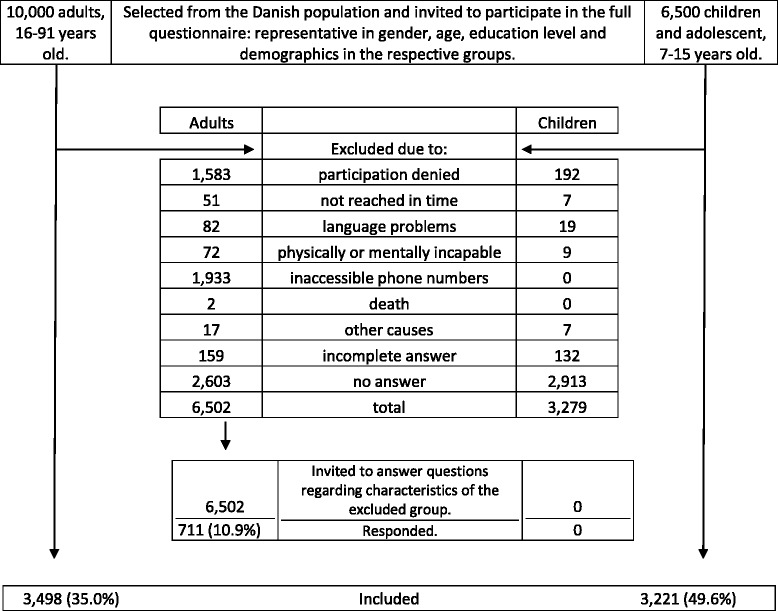
Recruitment Flowchart

A flow-chart is presented in Fig. 1.

### Questionnaire

The questionnaire, “Habits of Activities and Sports of the Danes”, consisted of 42 questions focusing on activity habits in every aspect. Similar versions of the survey had previously been distributed to the Danish population on eight previous occasions, starting in the sixties. However, the questionnaire distributed in 2016 was the first to include questions pertaining to sports injury. In the questionnaire, a sport-related injury, dichotomized into “yes” or “no”, was defined as “an injury sustained in relation to sport/exercise, which has prevented you from participating in sport/exercise for at least seven days, and/or which required contact with health professionals (doctor, physiotherapist or other)”, pooling single and multiple injuries together. The injury definition including “and/or” in regard to time loss and medical attention was chosen to keep the questionnaire as short as possible but still comparable to definitions used in other studies. The injury had to be present within the past 12 months, but could have been sustained before this period, however it was unknown whether prevention from activity or contact to health professionals, or both, was the reason.

The responders had to report gender, age, activity in general, activity-specific participation, injury status, and the specific activity causing the injury (the latter four categories focused on the past 12 months). Only activities with regular participation were mentioned, and multiple activities could be noted as having caused the injury. The length of 12 months was used to avoid the influence of seasonal variation in affecting changes to sports participation levels. The activity options in the questionnaire were based on knowledge from previous questionnaires, and included the most frequently played sports and activities in Denmark (IDAN Rapport 2011). An option to add an activity under the label “others” was included.

### Statistics

In the descriptive analysis, an injury prevalence (IP) was calculated representing the number of individuals reporting an injury, while the injury prevalence proportion (IPP) was the number of individuals reporting injuries divided by total number of respondents.

In the comparative analyses, prevalence proportion ratio was used to describe the association between injury prevalence proportion and age-groups, solely in the adult group. Prevalence proportion difference, by binomial regression, was used to compare the association between injury prevalence proportion and gender, both among adults and children.

An uneven distribution of multiple age and gender existed between responders and non-responders, including gender and age among adults, thus the adult group were analytically weighted so that each unit was inversely proportional to the variance of the observation. The weight reduced the discrepancy in age and gender in the responders compared with non-responders. No weight was used amongst children due to even response proportions between gender and age groups.

In the calculations focusing the “active” part of the sample, the persons who reported sports-related injuries within the past 12 months without reporting regular participation in any sports activity the past 12 months, were excluded, showing in the different number of injuries among all compared to the active part. This accounts for the calculations of injuries among “active”, “injuries across sports” and comparative analysis of age-groups, for both children and adults. The wording “active” in the present paper, refers to a participant answering to have been regularly participating in at least one activity within the past 12 months.

The data management and statistical analyses were handled in Stata (Stata/IC 14.0 for Mac, College Station, TX, USA), while Excel (Microsoft Excel for Mac, version 15.19.1) was used to compute Tables.

## Results

Of the 10,000 adults receiving a questionnaire, 3498 (35.0%) responded. After weighting the data, the sample consisted of 1719 males and 1779 females, of which 82.4% reported regularly being physically active, within the past year. A total of 642 (IPP = 18.4% (95% CI: 17.1%; 19.6%)) reported to have been injured at least once within the past 12 months. Males reported significantly more injuries (9.2%-points (95% CI: 6.7%-points; 11.8%-points)) than females, since the prevalence and prevalence proportion of injuries amongst males were 396 (23.1% (95% CI: 21.1%; 25.0%)) and amongst females 246 (13.8% (95% CI: 12.2%; 15.4%)).

Of the 2884 active adults, 620 persons reported an injury, equivalent to a prevalence proportion of 21.5% (95% CI: 20.0%; 23.0%). Among active adults, 27.4% (95% CI: 25.1%; 29.7%) and 15.9% (14.0%; 17.8%) of the males and females, respectively, reported injuries.

Of the 6500 children receiving a questionnaire, 3221 (49.6%) responded, of which 95.2% were regularly active within the last year. Injuries were presented in 621 (19.3% (95% CI: 17.9%; 20.6%)) children in the past 12 months. The injury prevalence proportion was similar amongst boys (19.5% (95% CI: 17.5%; 21.4%)) and girls (19.1% (95% CI: 17.1%; 21.0%)). Amongst physically active children, 19.9% (95% CI: 18.5%; 21.3%) reported an injury.

Both adults (Table 1) and children (Table 2) reported in which activities they regularly participated and in case of an injury, which activity had been related to the injury. Amongst adults, running was the sport contributing with the most injuries (n_inj_ = 198), followed by football (n_inj_ = 94) and strength training (n_inj_ = 89). Amongst children, football (n_inj_ = 235), handball (n_inj_ = 86) and gymnastics (n_inj_ = 66) were the sports with the highest prevalence of injuries.

**Table 1:** Injuries across sports among adults

Adults sport	participation			IP			IPP		
	a	95%	CI	n_inj_	95%	CI	n_inj_/a	95%	CI
running	1032	979	1085	198	173	223	0,19	0,17	0,22
football	248	218	278	94	79	109	0,38	0,32	0,44
strength	1047	994	1100	89	71	107	0,09	0,07	0,10
handball	87	69	105	34	25	43	0,39	0,29	0,49
badminton	193	167	219	24	15	33	0,12	0,08	0,17
gymnastics	295	263	327	18	10	26	0,06	0,03	0,09
hiking	896	845	947	18	10	26	0,02	0,01	0,03
road biking	275	244	306	15	8	22	0,05	0,03	0,08
tennis	79	62	96	14	7	21	0,18	0,09	0,26
mount. Biking	211	183	239	13	6	20	0,06	0,03	0,09
cross fit	144	121	167	12	5	19	0,08	0,04	0,13
skiing	267	236	298	10	4	16	0,04	0,01	0,06
aerobic	246	216	276	10	4	16	0,04	0,02	0,07
martial arts	56	41	71	9	4	14	0,16	0,06	0,26
golf	143	120	166	9	3	15	0,06	0,02	0,10
bike spinning	374	338	410	9	3	15	0,02	0,01	0,04
swimming	514	473	555	7	2	12	0,01	0,00	0,02
riding	61	46	76	7	2	12	0,11	0,03	0,19
skateboarding	24	14	34	7	3	11	0,29	0,11	0,47
basketball	25	15	35	6	2	10	0,24	0,07	0,41
volleyball	47	34	60	5	1	9	0,11	0,02	0,19
canoe / kayak	82	64	100	4	0	8	0,05	0,00	0,10
orientering	32	21	43	4	0	8	0,13	0,01	0,24
dance	154	130	178	4	0	8	0,03	0,00	0,05
hockey	27	17	37	3	–	–	0,11	–	–
parkour	13	6	20	2	–	–	0,15	–	–
yoga	303	270	336	2	–	–	0,01	–	–
sailing	35	23	47	2	–	–	0,06	–	–
boy scout	25	15	35	2	–	–	0,08	–	–
climbing	28	18	38	1	–	–	0,04	–	–
bowling	82	64	100	1	–	–	0,01	–	–
petanque	40	28	52	1	–	–	0,03	–	–
athletics	8	2	14	1	–	–	0,13	–	–
triathlon	23	14	32	1	–	–	0,04	–	–
roller skating	58	43	73	1	–	–	0,02	–	–
table tennis	35	23	47	1	–	–	0,03	–	–
wind- kite surf	15	7	23	1	–	–	0,07	–	–
hurting	105	85	125	1	–	–	0,01	–	–
handicap sport	9	3	15	1	–	–	0,11	–	–
open water	13	6	20	0	–	–	0,00	–	–
billiard	52	38	66	0	–	–	0,00	–	–
nordic walking	72	56	88	0	–	–	0,00	–	–
pilates	106	86	126	0	–	–	0,00	–	–
diving	33	22	44	0	–	–	0,00	–	–
rowing	26	16	36	0	–	–	0,00	–	–
wave surf	4	0	8	0	–	–	0,00	–	–
shooting	51	37	65	0	–	–	0,00	–	–
fishing	143	120	166	0	–	–	0,00	–	–
role playing	10	4	16	0	–	–	0,00	–	–

Participation, “a” number of participants

*IP*, injury prevalence, “n_inj_” number of injuries

*IPP*, injury prevalence proportion in different activities of adults, n_inj_/a the proportion

*CI*, confidence interval

Data are sorted by injury prevalence in descending order from highest to lowest

**Table 2:** Injuries across sports among children

Children sport	participation			IP			IPP		
	a	95%	CI	n_inj_	95%	CI	n_inj _/a	95%	CI
football	1177	1123	1231	235	208	262	0,20	0,18	0,22
handball	415	378	452	86	70	102	0,21	0,17	0,25
gymnastics	762	715	809	66	51	81	0,09	0,07	0,11
running	574	531	617	37	25	49	0,06	0,04	0,08
badminton	288	256	320	21	12	30	0,07	0,04	0,10
ridning	257	227	287	21	12	30	0,08	0,05	0,12
swimming	1132	1079	1185	17	9	25	0,02	0,01	0,02
dancing	381	345	417	16	8	24	0,04	0,02	0,06
strength training	377	341	413	15	8	22	0,04	0,02	0,06
trampoline	544	502	586	14	7	21	0,03	0,01	0,04
martial arts	208	181	235	14	7	21	0,07	0,03	0,10
basketball	81	64	98	10	4	16	0,12	0,05	0,20
kick scooter	478	438	518	9	3	15	0,02	0,01	0,03
parkour	100	81	119	8	3	13	0,08	0,03	0,13
skateboarding	196	169	223	7	2	12	0,04	0,01	0,06
boy scouting	345	311	379	7	2	12	0,02	0,01	0,04
tennis	117	96	138	6	1	11	0,05	0,01	0,09
athletics	65	49	81	5	1	9	0,08	0,01	0,14
hiking	190	164	216	4	0	8	0,02	0,00	0,04
bmx	45	32	58	4	0	8	0,09	0,01	0,17
volleyball	56	41	71	3	–	–	0,05	–	–
mountain biking	111	91	131	3	–	–	0,03	–	–
roller skating	268	237	299	3	–	–	0,01	–	–
aerobic teams	37	25	49	3	–	–	0,08	–	–
shooting	77	60	94	2	–	–	0,03	–	–
hockey	42	29	55	1	–	–	0,02	–	–
bike spinning	66	50	82	1	–	–	0,02	–	–
golf	43	30	56	1	–	–	0,02	–	–
table tennis	75	58	92	1	–	–	0,01	–	–
canoe / kayak / rowing	22	13	31	1	–	–	0,05	–	–
sailing	28	18	38	1	–	–	0,04	–	–
surfing	7	2	12	1	–	–	0,14	–	–
ice skating	75	58	92	1	–	–	0,01	–	–
road biking	34	23	45	0	–	–	0,00	–	–
role playing game	58	43	73	0	–	–	0,00	–	–
yoga	48	35	61	0	–	–	0,00	–	–
fishing	81	64	98	0	–	–	0,00	–	–

Participation, “a” number of participants

*IP* injury prevalence, “n_inj_” number of injuries

*IPP* injury prevalence proportion in different activities of adults, n_inj_/a the proportion

*CI* confidence interval

Data are sorted by injury prevalence in descending order from highest to lowest

Table 3 shows the injury prevalence proportion in different age-groups amongst adults and adolescents. These estimates focus only “physically active”. The prevalence proportion ratio is calculated with the youngest group, 16–19 years, as reference. The prevalence proportion of injured is continuously decreasing from the youngest to the oldest with only one plateau at 20–29 and 30–39.

**Table 3:** Comparative analysis of prevalence proportions in different age groups (adults only), with the 16–19 years as the reference group

Adults age	participants			IP			IPP			IPP-ratio		
	a	95%	CI	n_inj_	95%	CI	n_inj_ /a	95%	CI	IPP/IPP	95%	CI
16–19	197	170	224	76	63	89	0,39	0,32	0,45	1		
20–29	467	428	506	138	119	157	0,30	0,25	0,34	0,76	0,60	0,95
30–39	410	373	447	114	96	132	0,28	0,23	0,32	0,71	0,58	0,88
40–49	494	454	534	124	105	143	0,25	0,21	0,29	0,64	0,52	0,80
50–59	465	426	504	97	80	114	0,21	0,17	0,25	0,53	0,42	0,68
60–69	425	388	462	49	36	62	0,12	0,08	0,15	0,30	0,22	0,41
70+	426	389	463	23	14	32	0,05	0,03	0,08	0,14	0,09	0,22

Participation, “a” number of participants

*IP* injury prevalence, “n_inj_” number of injuries

*IPP* injury prevalence proportion in different activities of adults, n_inj_/a the proportion

*IPP*-ratio prevalence proportion-ratio, IPP/IPP the ratio between injury proportions

*CI* confidence interval

## Discussion

To our knowledge, no peer-reviewed articles have examined the injury prevalence and prevalence proportion of physically active persons in Denmark on a population level. The present study is therefore novel in the sense that contributes to the overall identification of the extent of the injury problem (Finch, 2006). The data presented in this study also suggest that sports injuries are frequent in Denmark, since a total of 18.4% of the adults and 19.3% of the children reported having had one or more injuries within the past 12 months, equal to either time lost with physical activity and/or contact to the health care system. We found more injuries amongst males than amongst females. The reason for this difference may be due to gender-specific differences in both physical aspects like anatomy but also psychologically aspects as mentality and behaviour when participating in certain sports. In addition, different preferences may exist between gender in the type of preferred physical activity and the exposure time of these activities, which, may be higher in males, which is detectable in the data set, and could be focused in future studies.

The consequences associated with sports-related injuries in Denmark are still largely unknown. For example, information about injury severity and recovery, potential absenteeism of work, use of therapeutic or surgical interventions, or time before returning to play (which may be equal to absenteeism from the health benefits of physical activity) are needed to understand the full impact from a population-level perspective.

According to Table 1, running was the sport which contributed to the most injuries (n_inj_ = 198) among adults, followed by football (n_inj_ = 94) and strength training (n_inj_ = 89). Accordingly, a reduction in the total number of sports injuries in the adult Danish population would benefit from a focus on preventing injuries sustained when running, playing football and engaging in strength training. Similarly, prevention of injuries in children and adolescents may require increased focus on preventing injuries associated with football (n_inj_ = 235), handball (n_inj_ = 86) and gymnastics (n_inj_ = 66) (Table 2). Importantly, no consequences in terms of absenteeism from work, surgery or time-to-recovery were reported. Therefore, some sports with a low injury prevalence, such as riding (7 injuries reported) amongst adults, may lead to severe injuries, such as spinal cord trauma etc., while the impact of injuries in other sports are less severe. This is not shown in the present data. Consequently, this is a major limitation that limited the possibility for evaluating the consequences of injuries across sports.

Table 3 shows the injury prevalence proportion ratio between age groups amongst active. The injury prevalence proportion continuously decrease from the youngest to the oldest, with only one plateau at 20–29 and 30–39. This observed trend could be explained by behavioural changes over time including changes to activity preference, as well as “the healthy athletes bias/effect” which is based on the rationale that only previously uninjured persons will continue to be active into older age.

The sample of the present study was recruited through CRS. Therefore, the 10.000 adults and 6.500 children and adolescents were representative of the population of Denmark in a number of variables, such as gender, age, education, ethnicity and demography. The response proportion among adults of 35% was lower than similar data collection in 2007 (43%) and 2011 (47%). Although the response proportion of 35% was low, the generalizability to the Danish population is presumably better than other studies examining the epidemiology of injury in specific target-populations such as elite athletes or members of certain sports clubs. However, the results in the present study may be affected by selection- and information bias. Owing to the response proportion of 35.0% amongst adults and 50% amongst children, it is reasonable to question whether the responders differed from the non-responders, (i.e. non-responders are hypothesized to be less active compared with responders). This selection problem was unsuccessfully handled by inviting the non-responders to answer a few questions describing their characteristics, but only 711 (10.9%) responded the phone call and the questions answered described the sub-sample insufficiently.

Amongst children, selection bias was less of a problem because of the higher proportion of persons responding, though the level of activity amongst responders may still be higher than non-responders. In summary, the selection problems addressed above may lead to selection bias amongst both adults and children leading to an overestimation of the proportion being injured in this sample altogether. The proportion of injured amongst active in general or across each sport may, however, be unaffected as it is unreasonable to believe that injury either motivates or prevents answering the questionnaire.

### Injury definition

The definition of injury used in the present study is almost similar to the consensus definition for runners, proposed by Yamato et al. (Yamato et al., 2015). It is well known that different injury definitions will find different injury prevalence in the same population. Similar definitions must be used before comparing results produced in epidemiological studies. In the present study, time-loss was used as the first component, since it is commonly used to define injury in many team sports given that it is easier to identify cases of injury (Clarsen & Bahr, 2014) In individual sports, however, it can be difficult to distinguish between reduced, modified, and/or not participating or participating with pain, thus “time-loss” will appear differently between sports and individuals, and 7 days of inhibition may not appear before severe physical complaint is present. Therefore, injury definition, as second component, also comprised a component: “and/or professional health care attention” which classifies a person as injured, irrespective of whether there has been activity time-loss. The use of “time-loss” has the disadvantage that it is very individual-dependent whether to stop training or just modify it. “Medical attention”, on the other hand, is very level-dependent, as people at high level of sports may see physiotherapists regularly to avoid losing valuable training time or important competitions, where less trained persons or beginners may take some time off instead of seeing health care. Thus the use of “and / or” will cover some of this discrepancy and may thereby give a more valid picture of the injury proportions.

### Perspective

Translating Research into Injury Prevention Practice (TRIPP) (Finch, 2006) is a framework to enhance prevention of sports-related injuries in a population. Injury surveillance studies must be conducted to identify if sports injuries are a public-health burden. The prevalence of injuries was demonstrated in the present study. Still, the consequences of these injuries require further investigation to fully understand the burden on public health.

The TRIPP framework highlights the importance of determining aetiology and mechanisms of injury. Bittencourt et al. (Bittencourt et al., 2016) promoted that the value of identifying single or multiple risk factors is limited in a prevention-perspective. In contrast, recognition of complex injury pattern as explanation of injury may be a new beneficial analytical approach (Bittencourt et al., 2016). If the injury pattern is recognized, next step is to develop preventive measures and test of the efficacy of the measures, first in ideal conditions, then implemented a in real world as guidelines for athletes and coaches (Soligard et al., 2016). Finally, additional epidemiological studies will be needed frequently to observe a potential effect of preventive interventions and a decline in injury prevalence on population level. Therefore, similar data collection on the prevalence and prevalence proportion of sports injuries in a sample representative of the Danish population will be conducted in the future. The next investigation made by the Danish Institute for Sports Studies will be in 2019/2020.

## Conclusion

According to the present study, sports injuries seem to be very frequent in Denmark, since a total of 18.4% of the adults and 19.3% of the children reported having had one or more injuries within the past 12 months, equal to either time lost with physical activity and/or contact to the health care system.
